# Lung, gastric and colorectal cancer mortality by occupation and industry among working-aged men in Japan

**DOI:** 10.1038/srep43204

**Published:** 2017-02-23

**Authors:** Hisashi Eguchi, Koji Wada, David Prieto-Merino, Derek R. Smith

**Affiliations:** 1 Department of Public Health, Kitasato University School of Medicine, Sagamihara, Japan; 2 Takemi Program in International Health, Department of Global Health and Population, Harvard T.H. Chan School of Public Health, Boston, USA; 3 Bureau of International Health Cooperation, National Center for Global Health and Medicine, Tokyo, Japan; 4 Faculty of Epidemiology and Population Health, London School of Hygiene & Tropical Medicine, London, United Kingdom; 5 Applied Statistical Methods in Medical Research Group, Catholic University of Murcia (UCAM), Murcia, Spain; 6 College of Public Health, Medical and Veterinary Sciences, James Cook University, Townsville, Australia

## Abstract

We examined occupational and industrial differences in lung, gastric, and colorectal cancer risk among Japanese men of working age (25–64 years) using the 2010 Japanese national survey data for occupation and industry-specific death rates. Poisson regression models were used to estimate the age-adjusted incident rate ratios by lung, gastric, and colorectal cancers, with manufacturing used as the referent occupation or industry. Unemployed Japanese men and those in manufacturing had an 8–11-fold increased risk of lung, gastric and colorectal cancer. The highest mortality rates for lung and colorectal cancer by occupation were “administrative and managerial” (by occupation) and “mining” (by industry). For gastric cancer, the highest mortality rate was “agriculture” (by occupation) and “mining” (by industry). By occupation; Japanese men in service occupations, those in administrative and managerial positions, those in agriculture, forestry and fisheries, and those in professional and engineering categories had higher relative mortality risks for lung, gastric, and colorectal cancers. By industry; mining, electricity and gas, fisheries, and agriculture and forestry had the higher mortality risks for those cancers. Unemployed men had higher mortality rates than men in any occupation and industry for all three cancers. Overall, this study suggests that for Japanese men, occupations and industries may be a key social determinant of health.

Lung, gastric, and colorectal cancer have now risen to become the leading three causes of cancer-related deaths among Japanese men^1^, and the Japanese government has therefore placed a high priority on further improving the cancer screening rate^2^. In order to maximize cancer prevention, the risk factors for these diseases warrant detailed investigation. Although tobacco use is the greatest risk factor for lung cancer; other factors such as age, radon exposure, environmental pollution, occupational exposure, sex, and pre-existing lung disease are also important contributors^3^. Risk factors for gastric cancer are known to include age, sex, tobacco smoking, radiation, and family history^4^; while risk factors for colorectal cancer include dietary and lifestyle factors such as lack of exercise, overeating, and eating red meat, processed meats, and perhaps; the consumption of refined carbohydrates^5^.

Occupationally-related hazards which contribute to the development of cancer represent an important avoidable cause of mortality^6^. Considerable evidence suggests that individuals working in coal and tin mining, metal processing (particularly steel and iron), and rubber manufacturing industries have increased risks of gastric cancer^7^,^8^. Occupational exposures play a significant role in lung cancer aetiology, and the risk of lung cancer is increased among workers employed in a number of industries and occupations such as aluminum production, coal production, painting and welding^9^. Previous studies have indicated increased risks of colorectal cancer risk within the Portland cement industry^10^, wood dust exposed workers^11^, and firefighters^12^.

Analysis of national datasets on occupational and industrial mortality contribute significantly to this endeavor because they incorporate data on either occupations or jobs and health outcomes over an entire population^13^. They are particularly useful in the assessment of diseases and injuries with high fatality rates; and also for diseases where fatality rates are lower. Occupation and industry classifications are commonly used as proxies for occupational exposure (post facto), to help identify and explore issues of worker morbidity and mortality from cancer^14^,^15^; particularly when this information is not available through other means.

For this study, we hypothesized that identifying occupations and industries with a higher risk of death due to lung, gastric and colorectal cancer would be a significant first step in elucidating some key social markers of health. In this regard, the Japanese Ministry of Health, Labour and Welfare produces an occupation-specific Vital Statistics Survey every 5 years^16^. The survey takes place in the same year as the national population census; with the government collecting information on occupations and industries to which deceased persons belonged at their time of death; and also records the cause of death from death certificates. Such data can potentially elucidate the magnitude of occupation and industry effects on health-related outcomes among working-age populations. To date, no studies have addressed occupational and industrial differences in lung, gastric, and colorectal cancer related deaths in Japan. The present study aimed therefore, to examine occupational and industry-related differences in lung, gastric, and colorectal cancer mortality rates among employed Japanese men aged 25 to 64 years.

## Methods

### Data sources

We obtained the most recent (2010) national dataset titles “Report of Vital Statistics: Occupational and Industrial Aspects” from the Japanese Ministry of Health, Labour, and Welfare. Occupation- and industry-specific death rates were calculated based on the 2010 national population census (also implemented at 5-year intervals on 1 October each year in Japan).

### Measurements

Japanese death certificate data includes the underlying causes of death and is completed by physicians based on the sequences of events leading to death, and coded according to the International Classification of Diseases (ICD), 10^th^ Revision^17^. Relevant codes for this study included lung cancer: C34.0–34.9, gastric cancer: C16.0–16.9, and colorectal cancer: CI8.0–18.9, C19, C20 and C21.0–C21.8. In the years that these statistics were collected in Japan, family members of deceased individuals were required to report the occupation and industry of the deceased person. Family members were given occupation and industry lists, and corresponding descriptions and definitions, and asked to select one occupational and one industry category. The occupation list included 11 occupations: administrative and managerial; professional; clerical; sales; services; security; agriculture, forestry and fisheries; manufacturing; transport; construction and mining; and carrying, cleaning and packaging. The industry list comprised 19 industries: agriculture; fisheries; mining; construction; manufacturing; electricity and gas; information; transport; wholesale and retail; finance; real estate and rental; research and professional services; accommodation and dining services; amusement services; education; medical and welfare; compound services; other service industries; and government. These categories are based on the International Standard Classification of Occupations^18^,^19^ and the International Standard Industrial Classification^20^,^21^. Detailed lists of occupations and industries used in Japanese national datasets have been published elsewhere^22^.

### Statistical analysis

We examined data for Japanese men who were aged 25–64 years in 2010, grouped into 5-year age intervals. Death rates for lung, gastric, and colorectal cancer in each age group by the occupation and industry categories defined in the Japanese national census. The numbers of employees from the national census in 2010^23^ was used as the denominator and the number of deaths as the numerator, to provide a Poisson distribution. We aggregated individual patient data in groups of industry and age to calculate the Poisson regression. Separate regression models estimated the incidence rate ratio (IRR) for lung, gastric and colorectal cancers in each occupation or industry category compared with the reference category of “manufacturing.” We then conducted separate regression models to estimate the IRR for lung, gastric, and colorectal cancers in each industry compared with the industry, “manufacturing”. Given that manufacturing has the largest number of categories, is traditionally the most advanced in taking occupational health measures, and also shares commonalities in both industry and occupation; manufacturing was selected as the reference category for this study.

Each occupation or industry model followed the same structure, as shown in the equation below. We included age, and the interaction term between the occupation or industry being tested and age, to allow for possible modifications of occupation or industry related effects at different ages. The logarithm of the expected number of lung, gastric or colorectal cancer deaths (*Y*) is a linear function of the logarithm of the exposed individuals (*N*), the occupation (or industry) being tested (*X*_i_), age, and the interaction between age and occupation (or industry). Age was coded as 0 for the reference category “25–29 years”, and then 1, 2, 3, and so on for older age groups. In each occupation category model, *X*_i_ was coded as 0 for the “manufacturing” category and 1 for the category being compared in the model. In the industry models, _i_ was coded as 0 for “manufacturing” and 1 for the industry being compared:


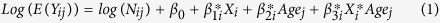


In this equation, exp(*β*_*0*_) estimates the rate in the occupation (or industry) reference group at age 25–29 years, exp(*β*_*1i*_) estimates the IRR between the occupation (or industry) being tested and the reference occupation (or industry) at age 25–29 years, exp(*β*_*2j*_) estimates the effect of age in the reference occupation (or industry), exp(*β*_*1i*_ *+* *β*_3i_*Age_*j*_) estimates the IRR between the occupation (or industry) being tested and the reference occupation (or industry) at a given age group “*j*”. The interaction coefficient *β*_*3i*_ allows modeling of a different effect due to occupation (or industry) by age. If the interaction coefficient was not significant, we re-estimated the model without the interaction term, and the effect of occupation (or industry) was captured by exp (*β*_*1i*_). Data were analyzed using STATA version 14 (StataCorp LP; College Station, TX, USA).

## Results

Tables 1 and 2 present the distribution of lung, gastric, and colorectal cancer mortality cases by occupation and industry in Japan. Lung cancer accounted for a total of 8,517 deaths among Japanese men aged 25–64 years during the study period. We excluded 1,269 men of unknown occupation category, and 1,440 men with no industry category; to give 7,248 lung cancer cases analyzed by occupation and 7,077 by industry. Gastric cancer accounted for a total of 6,319 deaths among Japanese men aged 25–64 years, from which we excluded 987 men without an occupation category, and 1,093 men without an industry category; resulting in 5,332 gastric cancer cases analyzed by occupation and 5,226 by industry. Colorectal cancer accounted for a total of 5,546 deaths among Japanese men aged 25–64 years, from which we excluded 838 men without an occupation category, and 908 men without an industry category, resulting in 4,708 colorectal cancer cases analyzed by occupation and 4,638 by industry.

Table 1 and Table 2 show the crude lung, gastric, and colon cancer mortality rates by occupation and industry, which are not age-adjusted. The largest number of deaths from lung, gastric, and colorectal cancers by occupational group was “professional and engineering”, and by industry; was “manufacturing”. The highest mortality rates for lung and colorectal cancer by occupation were “administrative and managerial” and by industry, “mining”. For gastric cancer, the highest mortality rate by occupation was “agriculture”, and by industry, “mining”. Unemployed men had higher mortality rates than men in any occupation and industry for all three cancers.

Table 3 indicates age-adjusted IRRs for lung, gastric, and colorectal cancers by occupation among Japanese males aged 25–64 years. When compared with the referent group (manufacturing), the unemployed, service, administrative and managerial, agriculture, forestry and fisheries and professional and engineering categories had higher relative mortality risks for these cancers. No interaction was found between age and occupation during this study.

Age-adjusted IRRs by industry for lung, gastric, and colorectal cancers are shown in Table 4. Mining, unemployed, electricity and gas, fisheries, and agriculture and forestry all had high risks for the three cancers. No interaction was found between age and industry.

## Discussion

This study examined the risk of premature mortality for lung, gastric, and colorectal cancer by occupation and industry among Japanese men aged 25–64 years. We found that unemployed Japanese men had the highest mortality risk for lung, gastric and colorectal cancer. When compared with manufacturing, occupations and industries with a relatively higher mortality by lung, gastric, and colorectal cancer were: “service”; “administrative and managerial”; “agriculture and fisheries”; “construction and mining”; “electricity and gas”; and “professional and engineering.” Common multiple risk factors for death due to lung, gastric, and colorectal cancer included smoking, work stress, and lifestyle.

Unemployment might incur the highest mortality risk for lung, gastric, and colorectal cancer among Japanese working-aged men in this study. The interaction works both ways, however, and several possible mechanisms may explain the relatively higher unemployment rates among cancer patients when compared to manufacturing workers. One mechanism may be that cancer patients might quit spontaneously following diagnosis or be fired by their employer if they do not meet the worker’s requirement in terms of physical and mental capacity due to the physical and/or mental side effects of their cancer treatment. It has been previously demonstrated that a significant proportion of cancer survivors may continue to experience levels of physical, emotional, and social problems that are elevated relative to the general population, with these issues sometimes persisting after the treatment period has ended^24^. Because the participants in the current (mortality) study had died from their cancer, it is conceivable that their symptoms might have affected their fitness for work, thereby leading to at least some period of unemployment prior to their death.

Service occupations had the highest mortality risk for lung, gastric, and colorectal cancers (all three) among Japanese working-aged men. One explanation relates to the hypothesis^25^,^26^ that nighttime shift work may be associated with an increase in cancer risk. Workers in service occupations are often required to work at night, for example, as these industries have the highest number of night-shift workers in Japan (28.6%) when compared with manufacturing (11.3%)^27^. Results from two previous mortality studies^28^,^29^ among male shift workers provided the first suggestions that an increased cancer risk was probably associated with night-shift work.

In a previous Japanese study, male mortality rates among administrative, and professional and engineering workers (who are usually of a higher social demographic) was shown to increase between 2000 and 2005 - more so than for other occupations that mostly comprised workers from lower social demographics^30^; a change could be attributed to the economic depression in Japan at that time^30^. In the current study, we found that the administrative and professional-and-engineering categories had higher risks for lung, gastric, and colorectal cancer in 2010, than categories with lower social class. These results may be explained by high psychological distress of administrative and professional occupations in Japan. Proposed biological mechanisms for the adverse effects of psychological distress on cancer development include neuroendocrine alterations in the hypothalamus-pituitary-adrenal axis regulating glucocorticoid release and the sympathetic nervous system regulating catecholamine levels^31^,^32^. In Japan, companies downsized their organizations after the economic recession in the 1990s; and consequently, the share of managers in the labor market decreased from 3.8% in 1990 to 2.6% in 2010 33. These changes in work structure could increase the overall responsibilities and job demands of managers compared with manufacturing^30^, which may increase the risk of those cancers through high psychological distress rates among male administrative and professional-and-engineering workers.

Men who work directly with natural resources in environments that contain dusty air (e.g., mining, and electricity and gas), have been shown to have higher risks of these cancers in Western countries^7^,^34^, similar to the current study. One possible explanation is the continuous exposure to higher levels of dust and chemical substances faced by workers in these occupations when compared to manufacturing. In Japan, the rate of dusty workplace environments has been shown to be higher in mining (50.1%) than in manufacturing (21.5%)^35^. The rate of workplaces with carcinogenic chemical substances is also known to be higher (24.4%) than in manufacturing (12.5%)^35^. The International Agency for Research on Cancer for example, has reported that there is an association with exposure to silica, some silicates, coal dust and lung, stomach, and colorectal cancer^36^. Further research on the role of responsible agents in these environments is necessary.

Workers in primary industries and occupations (e.g., agriculture and fisheries) had the higher mortality risk for lung, gastric, and colorectal cancers (all three) compared to manufacturing workers. The smoking rates, cancer screening rates and implementation rates of no smoking policies in the workplace do not differ between agriculture and fisheries and manufacturing^27^. Small-scale and family businesses are more common in primary industry than in manufacturing. It is reasonable to assume that small sized and family companies may have insufficient human resources for health activities when compared with large companies. It is important to note that Japanese companies with 50 or more employees are required by the Occupational Health and Safety Law to employ an occupational health physician. Therefore, a lack of physician supervision may be one reason why Japanese workers in primary industries are at a higher risk than their counterparts in manufacturing.

The current study may have incurred some limitations which should be considered. Firstly, the Japanese dataset only contains aggregate data by occupation (industry) and age category rather than individual-level data, and could not therefore adjust for individual-level variables (such as smoking or other lifestyle factors) that might confound the results. Secondly, for the vital statistics for death (numerator), families of the deceased persons selected their occupations and industries, which could have potentially led to misclassification in this regard. Thirdly, “Manufacturing” is a broad category and does not specify what types of exposures these workers actually incurred. Fourth, because we could not obtain actual work histories, we could not exclude the possibility that some workers developed the disease which ultimately killed them in their first job, but were not diagnosed at the time. Fifth, because this study was based on 2010 data only, we could not confirm the changes between 2010 and earlier years. Sixth, as census data was collected by self-administered questionnaires, numerator-denominator bias might also have occurred, considering the different information sources for the numbers of deaths, occupations and industries^37^. Such bias may occur if the distribution of populations across industries and occupations differs between the two data sources. Finally, in our analysis we have not compared the rates of cancer in working men with the rates in the general population. Although such a method might provide some interesting results, the research question of our current study was to compare cancer rates between industries in Japan, and not with the general population. It should be noted that working aged men are a subgroup that probably differ from the general population in many substantial ways (other than age). Crucially this data was collected within the limitations explained above that could introduce some biased comparisons with the general population. However, these biases would have occurred equally across all industries studied, therefore limiting its overall effect.

## Conclusion

In the current study, we identified differences of risks in mortality among lung, gastric, and colorectal cancer in Japanese working-age men, based on their occupations and industries. For Japanese men, occupations and industries may therefore be a key social determinant of health.

## Additional Information

**How to cite this article**: Eguchi, H. *et al*. Lung, gastric and colorectal cancer mortality by occupation and industry among working-aged men in Japan. *Sci. Rep.* **7**, 43204; doi: 10.1038/srep43204 (2017).

**Publisher's note:** Springer Nature remains neutral with regard to jurisdictional claims in published maps and institutional affiliations.

## Figures and Tables

**Table 1:** Characteristics of lung, gastric, and colon cancer mortality incidence by occupation among Japanese men aged 25–64 years in 2010.

			Lung cancer			Gastric cancer			Colorectal cancer		
	Total workers		Deaths		Incidence rate*	Deaths		Incidence rate*	Deaths		Incidence rate*
			n	(%)		n	(%)		n	(%)		n	(%)
Total	27,333,396	(100)	7,248	(100)	26.5	5,332	(100)	19.5	4,708	(100)	17.0
Administrative and managerial	661,738	(2.4)	359	(5.0)	54.3	231	(4.3)	34.9	209	(4.5)	31.6
Service	1,354,438	(5.0)	356	(4.9)	26.3	262	(4.9)	19.3	229	(4.9)	16.9
Agricultural, forestry and fishery	544,632	(2.0)	217	(3.0)	39.8	201	(3.8)	36.9	120	(2.6)	22.0
Security	725,493	(2.7)	52	(0.7)	7.2	33	(0.6)	4.5	26	(0.6)	3.6
Construction and mining	1,955,771	(7.2)	278	(3.8)	14.2	247	(4.6)	12.6	150	(3.2)	7.7
Transport and machine operation	1,464,903	(5.4)	199	(2.7)	13.6	146	(2.7)	10.0	120	(2.6)	8.2
Professional and engineering	3,734,063	(13.7)	480	(6.6)	12.9	404	(7.6)	10.8	362	(7.8)	9.7
Manufacturing	4,527,816	(16.6)	326	(4.5)	7.2	276	(5.2)	6.1	196	(4.2)	4.3
Clerk	3,614,473	(13.2)	252	(3.5)	7.0	188	(3.5)	5.2	163	(3.5)	4.5
Carrying, cleaning, packaging	1,252,228	(4.6)	79	(1.1)	6.3	54	(1.0)	4.3	37	(0.8)	3.0
Sales	3,513,746	(12.9)	324	(4.5)	9.2	222	(4.2)	6.3	158	(3.4)	4.5
Unemployed	3,984,095	(14.6)	4326	(59.7)	108.6	3068	(57.5)	77.0	2938	(63.2)	73.7

*Deaths per 100,000 workers.

**Table 2:** Characteristics of lung, gastric, and colon cancer mortality incidence by industry among Japanese men aged 25–64 years in 2010.

			Lung cancer			Gastric cancer			Colorectal cancer		
	Total workers		Deaths		Incidence rate*	Deaths		Incidence rate*	Deaths		Incidence rate*
			n	(%)		n	(%)		n	(%)		n	(%)
Total	26,974,828	(100)	7,077	(100)	25.2	5,226	(100)	18.8	4,638	(100)	17.1
Mining	13,460	(0.0)	32	(0.5)	237.7	19	(0.4)	141.2	10	(0.2)	74.3
Fisheries	68,412	(0.3)	39	(0.6)	57.0	45	(0.9)	65.8	20	(0.4)	29.2
Electricity and gas	211,138	(0.8)	93	(1.3)	44.0	74	(1.4)	35.0	49	(1.1)	23.2
Agriculture and forestry	449,240	(1.7)	204	(2.9)	45.4	192	(3.7)	42.7	124	(2.7)	27.6
Amusement services	553,089	(2.1)	95	(1.3)	17.2	62	(1.2)	11.2	57	(1.2)	10.3
Accommodations and dining services	776,134	(2.9)	132	(1.9)	17.0	93	(1.8)	12.0	78	(1.7)	10.0
Compound services	183,818	(0.7)	12	(0.2)	6.5	17	(0.3)	9.2	6	(0.1)	3.3
Construction	2,746,977	(10.2)	412	(5.8)	15.0	335	(6.4)	12.2	258	(5.6)	9.4
Government	1,252,730	(4.6)	114	(1.6)	9.1	80	(1.5)	6.4	80	(1.7)	6.4
Transport	1,951,734	(7.2)	255	(3.6)	13.1	194	(3.7)	9.9	169	(3.7)	8.7
Finance	571,999	(2.1)	71	(1.0)	12.4	53	(1.0)	9.3	51	(1.1)	8.9
Other services	1,346,970	(5.0)	178	(2.5)	13.2	103	(2.0)	7.6	91	(2.0)	6.8
Information	1,032,077	(3.8)	78	(1.1)	7.6	66	(1.3)	6.4	40	(0.9)	3.9
Medical and welfare	1,050,457	(3.9)	61	(0.9)	5.8	54	(1.0)	5.1	78	(1.7)	7.4
Manufacturing	5,158,183	(19.1)	492	(7.0)	9.5	408	(7.8)	7.9	312	(6.8)	6.0
Research and professional services	951,351	(3.5)	77	(1.1)	8.1	56	(1.1)	5.9	55	(1.2)	5.8
Real estate and rental	376,875	(1.4)	47	(0.7)	12.5	35	(0.7)	9.3	28	(0.6)	7.4
Education	863,779	(3.2)	47	(0.7)	5.4	44	(0.8)	5.1	35	(0.8)	4.1
Wholesale and retail	3,432,310	(12.7)	312	(4.4)	9.1	228	(4.4)	6.6	159	(3.5)	4.6
Unemployed	3,984,095	(14.8)	4326	(61.1)	108.6	3068	(58.7)	77.0	2938	(64.1)	73.7

*Deaths per 100,000 workers.

**Table 3:** Age-adjusted incident rate ratio (IRR) of mortality due to lung, gastric, and colorectal cancers by occupation among Japanese men aged 25–64 years in 2010.

Variable	Lung cancer		Gastric cancer		Colorectal cancer	
	IRR*	(95% Confidence Interval)	IRR*	(95% Confidence Interval)	IRR*	(95% Confidence Interval)
Unemployed	8.93	(6.84–11.7)	8.07	(6.00–10.8)	11.40	(8.09–16.1)
Service	3.28	(2.46–4.39)	2.96	(2.15–4.09)	3.65	(2.52–5.29)
Administrative and managerial	3.18	(2.38–4.26)	2.42	(1.74–3.38)	3.49	(2.40–5.07)
Agriculture, forestry and fisheries	2.11	(1.55–2.87)	2.49	(1.79–3.46)	2.11	(1.42–3.15)
Professional and engineering	1.99	(1.46–2.72)	2.01	(1.43–2.83)	2.48	(1.68–3.67)
Construction and mining	1.47	(1.05–2.04)	1.61	(1.13–2.29)	1.35	(0.87–2.08)
Sales	1.29	(0.92–1.81)	1.06	(0.72–1.56)	1.07	(0.68–1.69)
Transport and machine operation	1.14	(0.81–1.62)	1.05	(0.71–1.55)	1.31	(0.84–2.02)
Manufacturing	ref		ref		ref	
Clerical	0.82	(0.56–1.19)	0.74	(0.48–1.13)	0.87	(0.54–1.41)
Security	0.79	(0.54–1.16)	0.61	(0.39–0.96)	0.66	(0.39–1.11)
Carrying, cleaning, packaging	0.64	(0.42–0.96)	0.53	(0.33–0.85)	0.54	(0.31–0.94)

*When compared to individuals working in manufacturing.

**Table 4:** Age-adjusted incident rate ratio (IRR) of mortality due to lung, gastric, and colorectal cancers by industry among Japanese men aged 25–64 years in 2010.

Variables	Lung cancer		Gastric cancer		Colorectal cancer	
	IRR*	(95% Confidence Interval)	IRR*	(95% Confidence Interval)	IRR*	(95% Confidence Interval)
Mining	15.2	(11.9–18.8)	11.4	(8.8–14.7)	8.19	(6.08–11.0)
Unemployed	6.78	(5.35–8.59)	6.18	(4.75–8.04)	8.33	(6.19–11.2)
Electricity and gas	4.82	(3.77–6.12)	5.17	(3.96–6.75)	4.60	(3.07–5.75)
Fisheries	3.11	(2.41–4.01)	4.45	(3.40–5.83)	2.62	(1.88–3.65)
Agriculture and forestry	1.75	(1.33–2.32)	2.05	(1.52–2.76)	1.87	(1.32–2.65)
Amusement services	1.58	(1.19–2.09)	1.29	(0.93–1.79)	1.68	(1.18–2.40)
Accommodations and dining services	1.50	(1.13–2.00)	1.30	(0.94–1.80)	1.43	(0.99–2.07)
Information	1.46	(1.09–1.95)	1.43	(1.04–1.96)	1.05	(0.71–1.56)
Construction	1.12	(0.83–1.52)	1.12	(0.80–1.56)	1.15	(0.78–1.69)
Finance	1.23	(0.91–1.66)	1.08	(0.77–1.52)	1.28	(0.88–1.87)
Manufacturing	ref		ref		ref	
Transport	1.00	(0.74–1.37)	0.94	(0.66–1.34)	1.13	(0.77–1.66)
Real estate and rental	0.78	(0.56–1.09)	0.78	(0.54–1.12)	0.77	(0.50–1.17)
Government	0.99	(0.72–1.36)	0.77	(0.53–1.12)	1.07	(0.72–1.58)
Wholesale and retail	0.87	(0.63–1.20)	0.76	(0.52–1.10)	0.73	(0.47–1.13)
Medical and welfare	0.61	(0.43–0.88)	0.62	(0.42–0.92)	1.22	(0.83–1.78)
Research and professional services	0.69	(0.49–0.98)	0.62	(0.42–0.92)	0.81	(0.53–1.23)
Other services	0.89	(0.64–1.22)	0.62	(0.42–0.92)	0.80	(0.52–1.21)
Compound services	0.66	(0.46–0.94)	1.35	(0.98–1.86)	0.63	(0.40–0.99)
Education	0.47	(0.32–0.69)	0.49	(0.32–0.76)	0.56	(0.35–0.90)

*When compared to individuals working in manufacturing.
